# Screening and Identification of Six Serum microRNAs as Novel Potential Combination Biomarkers for Pulmonary Tuberculosis Diagnosis

**DOI:** 10.1371/journal.pone.0081076

**Published:** 2013-12-05

**Authors:** Xing Zhang, Jing Guo, Shufeng Fan, Yanyuan Li, Liliang Wei, Xiuyun Yang, Tingting Jiang, Zhongliang Chen, Chong Wang, Jiyan Liu, Zepeng Ping, Dandan Xu, Jiaxiong Wang, Zhongjie Li, Yunqing Qiu, Ji-Cheng Li

**Affiliations:** 1 Institute of Cell Biology, Zhejiang University, Hangzhou, China; 2 State Key Laboratory for Diagnosis and Treatment of Infectious Diseases, The First Affiliated Hospital, School of Medicine, Zhejiang University, Hangzhou, China; 3 Department of Radiology, Taizhou Hospital, Taizhou, China; 4 Department of Respiratory Medicine, Sixth of People’s Hospital of Shaoxing zhejiang, Shaoxing, China; 5 Department of Respiratory Medicine, Tongde Hospital of Zhejiang Province, Hangzhou, China; Sanjay Gandhi Medical Institute, India

## Abstract

**Background:**

It is very difficult to prevent pulmonary tuberculosis (TB) due to the lack of specific and diagnostic markers, which could lead to a high incidence of pulmonary TB. We screened the differentially expressed serum microRNAs (miRNAs) as potential biomarkers for the diagnosis of pulmonary TB.

**Methods:**

In this study, serum miRNAs were screened using the Solexa sequencing method as the potential biomarkers for the diagnosis of pulmonary TB. The stem-loop quantitative reverse-transcription polymerase chain reaction (qRT-PCR) assay was used to verify differentially expressed serum miRNAs. The receiver operating characteristic (ROC) curve and logistic regression model were used to analyze the sensitivity and specificity of the single miRNA and a combination of miRNAs for diagnosis, respectively. Using the predicted target genes, we constructed the regulatory networks of miRNAs and genes that were related to pulmonary TB.

**Results:**

The Solexa sequencing data showed that 91 serum miRNAs were differentially expressed in pulmonary TB patients, compared to healthy controls. Following qRT-PCR confirmation, six serum miRNAs (hsa-miR-378, hsa-miR-483-5p, hsa-miR-22, hsa-miR-29c, hsa-miR-101 and hsa-miR-320b) showed significant difference among pulmonary TB patients, healthy controls (*P*<0.001) and differential diagnosis groups (including patients with pneumonia, lung cancer and chronic obstructive pulmonary disease) (*P*<0.05). The logistic regression analysis of a combination of six serum miRNAs revealed that the sensitivity and the specificity of TB diagnosis were 95.0% and 91.8% respectively. The miRNAs-gene regulatory networks revealed that several miRNAs may regulate some target genes involved in immune pathways and participate in the pathogenesis of pulmonary TB.

**Conclusion:**

Our study suggests that a combination of six serum miRNAs have great potential to serve as non-invasive biomarkers of pulmonary TB.

## Introduction

Pulmonary tuberculosis (TB) caused by *Mycobacterium tuberculosis* (*Mtb*) is a chronic disease that can present serious threats to human health. According to the world health organization (WHO) report in 2012, an estimated one-third of the global population was infected with *Mtb*. There were 20 million estimated cases of TB, with 8.5–9.2 million new cases and 1.2–1.5 million deaths each year in the world [1]. Therefore, TB is still the leading cause of death by a single infectious agent. China, one of the countries most profoundly affected by pulmonary TB, has the world’s second largest TB epidemic, only behind India. Currently, in China, there are an estimated 4.51 million incident cases of pulmonary TB, including 1.3 million incident cases of active pulmonary TB, and 1.45 million new cases each year. About 1.3 million deaths occurred each year as a result of TB in China that accounted for 18% of the world’s population, and is more than the total of other infectious disease-related deaths [2].

The early diagnosis of pulmonary TB is important for control of the disease in the community [3]. But there are currently no validated biomarkers for diagnosis of pulmonary TB. So, new rapid, sensitive and efficient biomarkers or methods for pulmonary TB diagnosis are urgently needed to prevent the spread of pulmonary TB. This study is the first to screen serum miRNAs in pulmonary TB patients using Solexa sequencing, and verify differentially expressed serum miRNAs by qRT-PCR. The present study demonstrated that a combination of serum miRNAs may have great potential to serve as non-invasive biomarkers for detection of pulmonary TB. It is of great significance for diagnosis, treatment and prevention of pulmonary TB transmission.

## Materials and Methods

### Ethics Statement

This study was approved by the Ethics Committee of the Faculty of Medicine (Zhejiang University, China). Written informed consent was obtained from participants prior to their enrollment in the study. All legal guardians of included children gave written informed consent.

### Patients and Control Subjects

Serum samples were obtained from patients with pulmonary TB. The patients with the following criteria were included [4]: 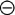 The patients diagnosed based on the clinical manifestations, bacterial culture, and radiographic findings. 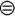 The patients with no major complications, such as chronic obstructive pulmonary disease (COPD), asthma, lung cancer, pneumonia, diabetes, and hypertension. 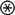 The patients with no family history of hereditary diseases. 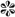 The patients with no past or present history of HIV infection, cancer, long-term hormone use, and organ transplantation. 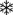 The patients who did not take anti-TB medication. There was no family history of hereditary diseases and low immune function in healthy controls. There were no significant differences in age and gender between pulmonary TB patients, the differential diagnosis group and healthy controls.

A total of 128 subjects with pulmonary TB (male 86, female 42), aged 16–64 years (mean age 43 years) were recruited from the Sixth Hospital of Shaoxing, Zhejiang province, China. In addition, 108 healthy controls (male 76, female 32) aged 24–67 years (mean age 44 years) were recruited from the Zhejiang Hospital between October 2010 and July 2012.

A total of 90 subjects in the differential diagnosis group (30 COPD patients, 30 lung cancer patients and 30 pneumonia patients), confirmed clinically after eliminating pulmonary TB disease, were recruited from the Zhejiang Cancer Hospital, Zhejiang Xinhua Hospital, Zhejiang Provincial People’s Hospital and Zhejiang Tongde Hospital between November 2011 and July 2012 (Table 1). A multiphase, case-control study was designed to identify serum miRNAs as surrogate makers for pulmonary TB (Figure S1).

**Table 1:** Characteristics of study participants with pulmonary TB, healthy controls, lung cancer, pneumonia and COPD.

Characteristic	Pulmonary TB		Healthy Controls		Differential Diagnosis Patients		
	Solexa sequencingtraining set	validationset	Solexa sequencingtraining set	validationset	LungCancer	Pneumonia	COPD
Total Number	20	108	20	88	30	30	30
Gender (male/Female)	13/7	73/35	11/9	65/23	23/7	16/14	13/17
Ethnicity (Han Chinese)	20	108	20	88	30	30	30
Age (years, average; range)	41 (30–64)	45 (14–62)	37 (29–65)	48 (24–67)	57 (30–69)	43 (23–64)	68 (57–80)
Pulmonary TB	20	108	NA	NA	NA	NA	NA
TST (Positive/Negative)	18/2	99/9	NA	NA	NA	NA	NA
BCG vaccination (Yes/No)	18/2	97/11	20/0	88/0	30/0	30/0	30/0
Smear test (Positive/Negative)	8/10	87/21	NA	NA	NA	NA	NA
History of smoking (Yes/No)	11/9	43/65	NA	NA	NA	NA	NA
Sputum culture-proven	10	56	NA	NA	NA	NA	NA
Chest X-ray and CT-proven	20	108	NA	NA	NA	NA	NA
HIV-negative	20	108	20	108	30	30	30

NA, Not applicable; TB, Tuberculosis; COPD, chronic obstructive pulmonary disease; TST, Tuberculosis Skin Test; BCG, Bacille Calmette-Guerin vaccine; HIV, Human immunodeficiency virus.

Fasting early morning blood samples were collected from the patients and controls in 3.0 ml tubes. Within 4 h of collection, these samples were centrifuged at 1,200 g for 10 min at 4°C to spin down the blood cells, and the supernatant was transferred into microcentrifuge tubes, followed by a second centrifugation at 12,000 g for 10 min at 4°C. The supernatant was transferred to RNase/DNase-free tubes and stored at −80°C.

### RNA Extraction

Total RNA was isolated from the serum using a DNA extraction kit (Qiagen miRNeasy® Mini kit, No. 217004, Germany) according to the manufacturer’s instructions. Briefly, 200 µl of human serum was used to extract total RNA. Each sample was eluted in 30 µl of RNAse-free water. RNA quantity and purity was measured using a NanoDrop spectrophotometer (ND-1000; ThermoScientific, DE, USA). In solution, pure RNA has an A260/A280 ratio of 1.8–2.0. If the absorbance ratio was significantly less than 1.8, the nucleic acid was probably contaminated with protein. The total RNA concentration was 5–10 ng/µl.

### Solexa Sequencing

The total RNA of 20 pulmonary TB patients and 20 healthy controls was purified directly for Solexa sequencing analysis using an Illumina Genome Analyzer (Illumina, San Diego, CA, USA) according to the manufacturer’s instructions [5]. The total RNA concentration of samples for Solexa sequencing were at least more than 5 ng/µl (≥100 ng RNA) containing less protein and salt ion impurities. After PAGE purification of small RNA molecules (<30 bp) and ligation of a pair of Solexa adaptors to their 5′ and 3′ ends, the small RNA molecules were amplified using the adaptor primers for 17 cycles. Fragments of around 90 bp in length (small RNA+adaptors) were isolated from agarose gels. The purified DNA was directly used for cluster generation and sequencing analysis using the Illumina Genome Analyser (Illumina, San Diego, USA), according to the manufacturer’s instructions [6]. The 35 bp sequences were obtained from Solexa sequencing. The image files generated by the sequencer were processed to produce digital-quality data. The primary analysis included that the original reads removed the adaptors, the low-quality reads and decontamination, and were computed distribution length of sequences. Then the high-quality sequencing reads were obtained. The sequence obtained from the primary analysis, were classified and noted, then each component and the information from the sample could be obtained. After a bioinformatics analysis, the remaining miRNAs were searched against the Sanger miRBase (version15.0) [7] to identify conserved, known miRNAs and novel miRNA. Then, the differences in the quantities of the known miRNAs between the two groups were determined by comparing the log2 ratio of copies (copies of pulmonary TB/copies of healthy controls).

### Solexa Sequencing Data Submission

The raw data of a deep-sequencing was submitted to the “NIH Short Read Archive” database, and is available under the following accession numbers: SRP029907, SRS479660, SRX349068, SRR978406, SRX349069, SRR978407.

### MiRNAs Quantification by qRT-PCR

A multiphase, case-control study was designed to identify serum miRNAs as surrogate makers for pulmonary TB. In the training set, miRNAs were measured in the serum samples from 20 pulmonary TB patients and 20 healthy controls. Meanwhile, in the validation set, miRNAs were measured in the serum samples from 108 pulmonary TB patients, 88 healthy controls, and 90 differential diagnosis groups (including 30 pneumonia, lung cancer and 30 COPD patients). Reverse transcription-specific stem-loop primers and the primers for fluorescent quantitative PCR were designed according to the previously described methods [8]. The design principles for miRNAs-specific primers were that the 3′ end of universal stem-loop primer sequences were added to the 3′ end of corresponding reverse complementary mature miRNAs sequences by using the same 44 nt stem-loop sequence (5′-GTCGTATCCAGTGCAGGGTCCGAGGTATTCGCACTGGATACGAC-3′) for all RT primers. The universal reverse primer sequence: 5′-AGTGCAGGGTCCGAGGTATT-3′. The miRNA-specific forward primer sequences are shown in Table S1.

In brief, serum RNA containing miRNA was polyadenylated by poly (A) polymerase and reverse transcribed to cDNA using Fermentas RevertAid™ First Strand cDNA Synthesis Kit (K1622, Canada) according to the manufacturer’s instructions. After total RNA isolation, reverse transcription (RT) was done in a reaction volume of 5.0 ml, which contained 2.5 µl of digested RNA preparations, 0.5 µl of RNase inhibitor RRI, 0.5 µl of dNTPs (10 mM each), 1.0 µl of 5×M-MuLV buffer, 0.5 µl of miRNA-specific RT primer (2 µM), 0.25 µl M-MuLV reverse transcriptase. Using the two-step method, the first step was 65°C for 5 min, ice for 10 min, and the second step was 42°C, 60 min, 70°C for 15 min. After the reaction, the cDNA were stored at −80°C refrigerator to spare.

SYBR green qRT-PCR assay (TaKaRa SYBR® *Premix Ex* Taq™ Tli RnaseH Plus, DRR420A, Dalian, China) was used for miRNA quantification in serum samples. Briefly, the total volume of reaction mixture was 20 µl, including 10 µl SYBR system Mix, 0.4 µl 50*ROX refenence dye, 0.8 µl forward primer (10 µM) and reverse prime (10 µM) respectively, 1 µl cDNA and 7 µl ddH_2_O. Then, cooled the plate and centrifuged plate briefly in Mini Plate Spinner. The PCRs were carried out with incubation at 95°C for 30 s followed by 40 cycles of 95°C for 5 s and 60°C for 31 s using an ABI PRISM 7300 detection system (Applied Biosystems, Foster City, CA, USA). Each sample was run in triplicate.

### Data Processing and Statistical Analysis

For qRT-PCR data, the expression level of miRNA was normalized to miR-16 that was stable in serum samples [9]. The mean for miR-16 was the same across all cohorts. The CT is defined as the fractional cycle number at which the fluorescence exceeds the defined threshold. The relative expression levels of each target miRNAs (Log2 relative level) were calculated according to the difference in CT values between the target miRNAs and miR-16 by using the 2^−ΔΔCT^ method [10]. −ΔΔCT = (CT miRNA - CT miR-16) patients - (CT miRNA - CT miR-16) controls. The miR-16 CT values were lower than the means of the other miRNA per cohort. The experiment set up a negative control that the templates were ddH_2_O. Each sample was run in triplicate. Statistical analysis was performed with GraphPad Prism 5 software (Graphpad Software Inc., San Diego, CA, USA). Data are presented as the mean ± SD (standard deviation). Nonparametric Mann-Whitney test was used to compare difference in serum miRNA concentration between pulmonary TB patients and healthy controls. A *P*-value <0.05 was considered statistically significant. For each miRNA, a receiver operating characteristic (ROC) curve was generated. The area under curve (AUC) value and 95% confidence intervals (CI) were calculated to determine the specificity and sensitivity of diagnosis of pulmonary TB. To increase the diagnostic accuracy of a combination of serum miRNAs, multiple logistic regression analysis was carried out. ROC curve and logistic regression model were calculated by using MedCalc Software (Version 12.3.0, Belgium).

### MiRNA-Gene Network Construction

TargetScan (http://genes.mit.edu/targetscan/index.html) and PicTar (http//pictar.bio.nyu.edu) database were used to predict genes targeted by the obtained miRNAs. Target genes associated with pulmonary TB were screened using NCBI (http://www.ncbi.nlm.nih.gov/pubmed/) database and Cytoscape Software (Cytoscape Software, Version 2.8.2, Seattle, USA) [11]. The miRNA-Gene-Network was constructed using Cytoscape Software to analyze the interactions between miRNA and genes [12]. The molecular functions of target genes were based on the Gene Ontology (http://www.geneontology.org/) and Kyoto Encyclopedia of Genes and Genomes (http://www.genome.jp/kegg/).

## Results

### Solexa Sequencing of Serum miRNAs between Pulmonary TB and Healthy Controls

The miRNA concentration of serum samples from 20 pulmonary TB patients and 20 healthy individuals were examined by Solexa sequencing. Table 2 and Table 3 summarized that the major components of small RNAs and the number of miRNAs from pulmonary TB patients and healthy controls by Solexa sequencing analysis. Among the serum miRNAs detected by Solexa sequencing, 233 miRNAs were found in pulmonary TB patients and 253 miRNAs were found in healthy controls (Table 2). Ninety-one serum miRNAs with significantly altered expression were identified between pulmonary TB patients and healthy controls, of which 44 were over-expressed and 47 were under-expressed in pulmonary TB patients, compared to the healthy controls. New miRNAs precursors were predicted, 13 miRNAs in pulmonary TB patients and 19 miRNAs in healthy controls were discovered (Table S2).

**Table 2:** The categories of small RNAs in pooled serum samples from pulmonary TB patients and healthy controls by Solexa sequencing analysis.

	Healthy controls		Pulmonary TB	
	Unique sRNAs	(%)	Unique sRNAs	(%)
Total (matchgenome)	215944	100%	232793	100%
exon-antisense	38	0.02%	52	0.02%
exon-sense	199	0.09%	224	0.10%
intron-antisense	178	0.08%	185	0.08%
intron-sense	256	0.12%	266	0.11%
miRNA	253	0.12%	233	0.10%
rRNA	17095	7.92%	20868	8.96%
repeat	1417	0.66%	1550	0.67%
scRNA	653	0.03%	469	0.20%
snRNA	676	0.31%	757	0.33%
snoRNA	87	0.04%	71	0.03%
srpRNA	2	0.00%	1	0.00%
tRNA	8713	4.03%	11417	4.90%
unannotated	186377	86.31%	196700	84.50%

**Table 3:** The number of miRNAs in pooled serum samples from pulmonary TB and healthy controls by Solexa sequencing analysis.

Sample	Number of miRNA	Number of miRNA*	Number of precursors	Number of hairpin-matched precursors (unique)	Number of hairpin-matched precursors (total)
Known miRNA in miRBase	1539	194	1424	–	–
Healthy controls	90	13	111	254	4438956
Pulmonary TB	82	11	102	236	3922091

* the opposite arm of the precursor.

Differentially serum miRNAs were considered significant candidates for further study only when they fulfilled the following criteria [13], [14]: 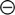 having at least 10 copies of miRNA expression; 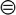 mean fold change >1.5 or ratio <0.5, and with a high percentage; 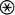 *P* values <0.01; 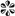 pulmonary TB associated miRNAs previously reported in the literature. Based on these criteria, 15 candidate serum miRNAs (10 miRNAs were up-regulated and 5 miRNAs were down-regulated in pulmonary TB) were chosen for qRT-PCR validation (Table S3).

### Differential Expression of miRNAs between Pulmonary TB and Healthy Controls by qRT-PCR Analysis

Fifteen candidate miRNAs were verified by qRT-PCR analysis. In the training set, miRNAs were measured in a separate set of individual serum samples from 20 pulmonary TB and 20 healthy controls (the same with Solexa sequencing). The miRNAs with a mean fold change (pulmonary TB/healthy controls) ≥1.5 or ≤1.0 and *P*<0.01 were selected for further analysis. Moreover, miRNAs with a CT value >35 and a detection rate <75% in either pulmonary TB or healthy controls were excluded from further analysis. The analysis generated a list of six miRNAs (hsa-miR-378, hsa-miR-483-5p, hsa-miR-22, hsa-miR-29c, hsa-miR-101, and hsa-miR-320b) that showed a difference expression between pulmonary TB and healthy controls in the training set (Table 4).

**Table 4:** Differentially expressed serum miRNAs in pulmonary TB compared to the healthy controls in both the training set and the validation set.

miR-names	Training set		Validation set	
	Fold-change	*P*-value	Fold-change	*P*-value
Up-regulation in pulmonary TB				
hsa-miR-378	1.90824	0.0071**	2.92755	<0.0001***
hsa-miR-483-5p	2.24424	0.0106*	1.79118	0.0010**
hsa-miR-22	1.79089	0.0119*	3.38051	0.0168*
hsa-miR-29c	1.71563	0.0007***	3.43867	<0.0001***
Down-regulation in pulmonary TB				
hsa-miR-101	0.13027	<0.0001***	0.69259	0.0010***
hsa-miR-320b	0.82879	0.0253*	0.68944	<0.0001***

*** *P*<0.001,

** *P<*0.01,

* *P*<0.05.

**Figure 1:**
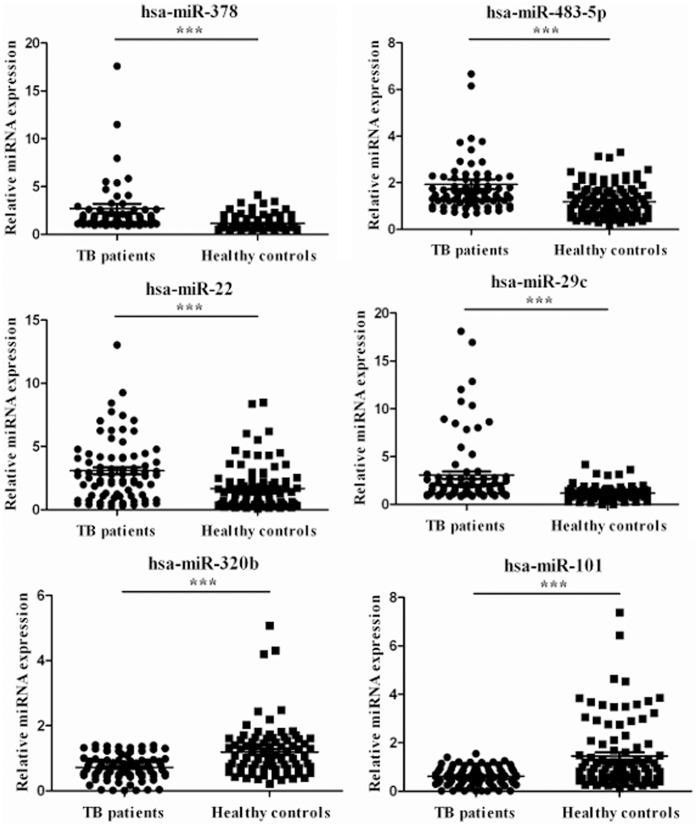
Detection of pulmonary TB patients with six serum miRNAs by qRT-PCR assay. Serum miRNA expression of six miRNAs was measured in 128 pulmonary TB patients and 108 healthy controls (in both the training and validation set). We analyzed the expression of six miRNAs (hsa-miR-378, hsa-miR-483-5p, hsa-miR-22, hsa-miR-29c, hsa-miR-320b and hsa-miR-101) selected from the Solexa sequencing data by using qRT-PCR. The 2^−ΔΔCT^ method was used to normalize the relative gene expression data in the qRT-PCR assay. hsa-miR-16 was set as the reference gene. The miRNA expression value in one subject with healthy controls was normalized to 1. Statistical analysis was performed using the nonparametric Mann-Whitney test. ***P<0.001, **P<0.01, *P<0.05. TB, tuberculosis.

In the validation set, these six miRNAs were further examined by qRT-PCR in a large cohort consisting of 108 patients with pulmonary TB and 88 healthy controls. Consistent with the results from the training set, the serum concentrations of the six miRNAs were significantly different between pulmonary TB patients and healthy controls in the validation set (Table 4). The qRT-PCR analysis was consistent with Solexa sequencing data: the expression of hsa-miR-378 (*P*<0.0001), hsa-miR-483-5p (*P = *0.0010), hsa-miR-22 (*P = *0.0168) and hsa-miR-29c (*P*<0.0001) were present in higher abundance while, hsa-miR-101 (*P = *0.0010) and hsa-miR-320b (*P*<0.0001) were present in lower abundance in the pulmonary TB serum than that of the healthy controls in both the training and validation set (Figure 1).

### Detection of Serum miRNAs from Pulmonary TB and Differential Diagnosis Groups by qRT-PCR Analysis

**Figure 2:**
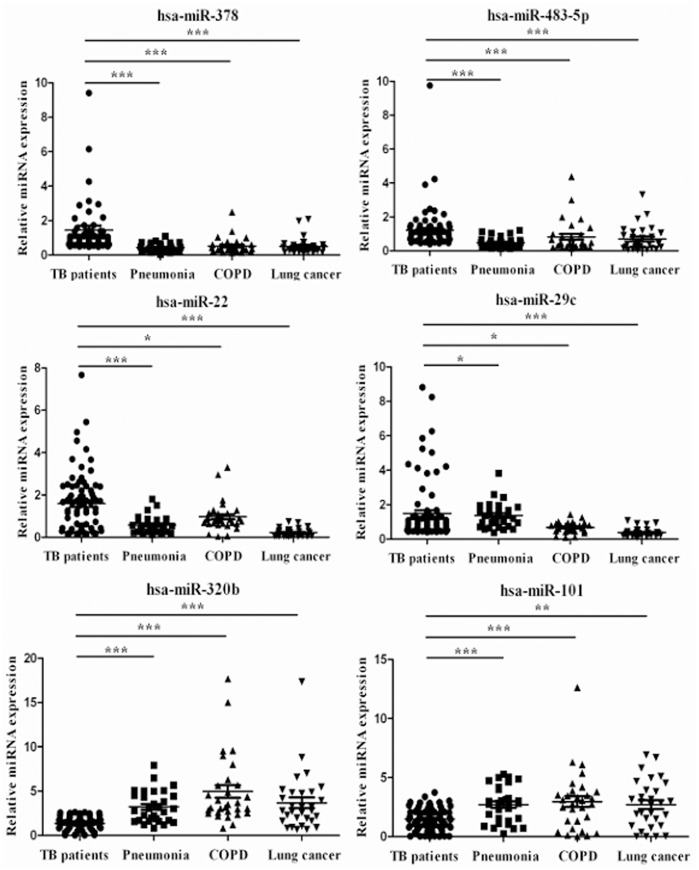
The qRT-PCR assay validation of miRNA expression levels in samples from pulmonary TB patients versus pneumonia, COPD or lung cancer patients. We analyzed the expression of six serum miRNAs (hsa-miR-378, hsa-miR-483-5p, hsa-miR-22, hsa-miR-29c, hsa-miR-320b and hsa-miR-101) in 128 pulmonary TB patients versus 30 pneumonia, 30 COPD or 30 lung cancer patients, respectively. The 2^−ΔΔCT^ method was used to normalize the relative gene expression data in the qRT-PCR assay. Hsa-miR-16 was set as the reference gene. The miRNA expression value in one subject with pulmonary TB was normalized to 1. Statistical analysis was performed using the nonparametric Mann-Whitney test. ***P<0.001, **P<0.01, *P<0.05. TB, tuberculosis; COPD, chronic obstructive pulmonary disease.

The qRT-PCR validation were carried out to quantify the differential expression levels of the six miRNAs in the samples from 128 pulmonary TB patients and 90 differential diagnosis groups (including 30 pneumonia, lung cancer and COPD patients, respectively). The data showed that the six miRNAs were significantly different between pulmonary TB and other three groups (*P*<0.05) (Figure 2).

### ROC Curve Analysis of a Combination of Six miRNAs

**Figure 3:**
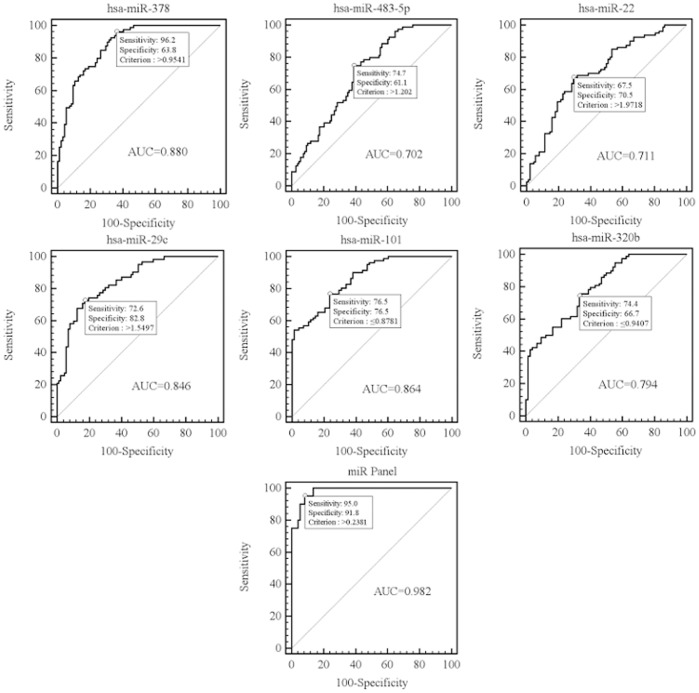
Receiver operating characteristic (ROC) curves for the ability of serum levels of the selected six individual miRNAs (A–F) and the combined six-miRNA panel (G) to differentiate pulmonary TB patients (n = 128) from healthy control subjects (n = 108) in both the training and validation set. TB, tuberculosis.

ROC curves constructed to compare the relative concentrations of the six miRNAs for pulmonary TB patients and healthy controls yielded the following AUCs: hsa-miR-378, 0.880 (*P*<0.0001, 95% CI = 0.822–0.925); hsa-miR-483-5p, 0.702 (*P*<0.0001, 95% CI = 0.631–0.766); hsa-miR-22, 0.711 (*P*<0.0001, 95% CI = 0.637–0.779); hsa-miR-29c, 0.846 (*P*<0.0001, 95% CI = 0.778–0.900); hsa-miR-101, 0.864 (*P*<0.0001, 95% CI = 0.798–0.914); hsa-miR-320b, 0.794 (*P*<0.0001, 95% CI = 0.720–0.856). Using the optimal cutoff value, we obtained the following sensitivity and specificity values: hsa-miR-378, 96.2% and 63.8%, respectively; hsa-miR-483-5p, 74.7% and 61.1%, respectively; hsa-miR-22, 67.5% and 70.5%, respectively; hsa-miR-29c, 72.6% and 82.8%, respectively; hsa-miR-101, 76.5% and 76.5%, respectively; and hsa-miR-320b, 74.4% and 66.7%, respectively (Figure 3). The results showed that a single serum miRNA for the diagnosis of pulmonary TB had lower sensitivity and poor specificity.

Multivariate logistic regression analysis in ROC curve was used to identify the best combination of six miRNAs to diagnose pulmonary TB. The following regression equation was built: Logit (p) = −11.4809+3.47232* (hsa-miR-378)+1.99529* (hsa-miR-483-5p)+1.94199* (hsa-miR-29c)+1.16943* (hsa-miR-22)−1.52372* (hsa-miR-320b)−2.09126* (hsa-miR-101) (Table 5). In multiple logistic regression analysis of a combination of six miRNAs, the resulting ROC curve had an AUC value of 0.982 (*P*<0.0001, 95% CI = 0.929–0.998), which reflects strong separation between pulmonary TB patients and healthy controls. The sensitivity was 95.0% and the specificity was 91.8%, which were more than a single serum miRNA in both the training and validation set (Figure 3). If the AUC of a combination of 6 serum miRNAs was more than 0.9, the accuracy for diagnosis of pulmonary TB was higher.

**Table 5:** Logistic regression analyses of a combination of six serum miRNAs for the diagnosis of pulmonary TB in both the training set and the validation set.

Logistic regression analyses			
*Coefficients and standard errors*			
Variable	Coefficient	Std. error	*P* value
hsa-miR-378	3.47232	1.31956	0.0085
hsa-miR-483-5p	1.99529	0.95722	0.0371
has-miR-29c	1.94199	1.07872	0.0718
has-miR-22	1.16943	0.46606	0.0121
has-miR-320b	−1.52372	1.78138	0.3924
has-miR-101	−2.09126	1.97946	0.2907
Constant	−11.4809		
Logit (p) = −11.4809+3.47232* (hsa-miR-378)+1.99529* (hsa-miR-483-5p)+1.94199* (hsa-miR-29c)+1.16943* (hsa-miR-22)−1.52372* (hsa-miR-320b)−2.09126* (hsa-miR-101)			
***Odds ratios and 95% confidence intervals***			
**Variable**	**Odds ratio**	**95% CI**	
hsa-miR-378	32.2114	2.4254 to 427.7978	
hsa-miR-483-5p	7.3543	1.1265 to 48.0112	
has-miR-29c	6.9726	0.8417 to 57.7592	
has-miR-22	3.2202	1.2917 to 8.0279	
has-miR-320b	0.2179	0.0066 to 7.1548	
has-miR-101	0.1235	0.0026 to 5.9803	

### Construction of miRNA-Gene-Network

**Figure 4:**
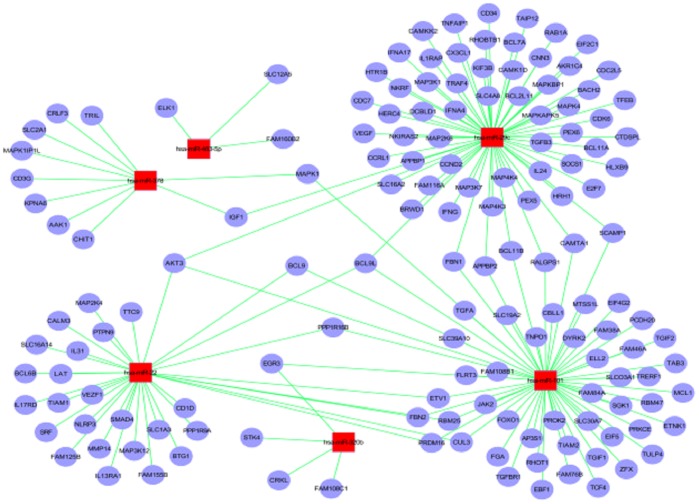
The miRNA-gene-network for the association between miRNAs and genes related to pulmonary TB. The miRNA-gene-network was constructed using the gene expression data and the predicted interactions in the TargetScan miRNA database. Circles represent genes and squares represent miRNAs; their relationship is represented by one edge. The center of the network represents the degree.

The miRNA-gene-network was constructed based on the data sets consisting of miRNA-target gene binding information and expression profiles of miRNAs and mRNAs (Figure 4). Totally 3444 target genes were regulated by six miRNAs. By the analysis of software and database, 176 target genes were associated with pulmonary TB. Hsa-miR-29c had the highest degree of regulation with 70 target genes, followed by hsa-miR-101 with 57 target genes, hsa-miR-22 with 32 target genes, and hsa-miR-378 with 10 target genes. Hsa-miR-483-5p and hsa-miR-320b showed the minimum degree of regulation with only 3 and 4 target genes, respectively.

Among 176 target genes associated with pulmonary TB, most genes (134) were targeted by only one miRNAs in the network (IFNG was only targeted by hsa-miR-29c). The remaining 42 genes were regulated by multi-miRNAs: 15 genes were regulated by 2 miRNAs (MAPA1 was targeted by hsa-miR-378 and hsa-miR-101), and 2 genes were regulated by 3 miRNAs. The AKT3 (threonine-protein kinase) and BCL9L (B-cell CLL/lymphoma 9-like) genes were regulated by 3 miRNAs. Analysis of GO for 176 genes, based on the molecular function and KEGG database, showed that most encoded proteins with transcription regulator activities and protein binding functions such as focal adhesion, MAPK signaling, Wnt signaling, insulin signaling and TGF-beta signaling were involved in cellular growth, movement, and proliferation.

## Discussion

*Mtb* is transmitted through droplet infection, and affects lives of people in close contact with TB patients or asymptomatic undiagnosed subjects in the society [4]. Rapid and accurate diagnosis is essential for the control of TB spread and adequate antimicrobial therapy [15]. There are a variety of methods in clinical diagnosis of pulmonary TB [16]. Sputum smear provides rapid results and is widely used in clinical laboratories, but this conventional method shows low positive rate of 20% to 30%. The gold standard of pulmonary TB diagnostics is confirmation with organism growth in selective media, but this culture in clinical specimens requires long incubation time (4–8 weeks) for slow growth of mycobacteria with positive rate of 30% to 40%. In the early stage of TB infection, imaging appearances are nonspecific and it may be difficult to distinguish pulmonary TB from other lung diseases, for example, cavitary pulmonary TB can be misdiagnosed as lung abscess, and diffuse bilateral pulmonary TB can be misdiagnosed as interstitial lung disease. Bacille Calmette–Guérin vaccination can cause a false positive reaction to a TB skin test. Immunological tests can detect active TB with an accuracy rate of about 50% because the antigens for *Mtb* are complex and have different antibodies spectrum due to differences in immune background and physical condition of individuals. Therefore, new biomarkers for pulmonary TB diagnosis are urgently needed.

Recently, serological markers for pulmonary TB have been intensively studied. Agranoff et al. [17] found two serum markers (serum amyloid A protein and transthyretin) in the African population of TB patients by using matrix assisted laser desorption/ionization - time of flight - mass spectrometry (MALDI-TOF-MS). But the authors did not establish the differential diagnosis groups, and verified two serum markers in a large sample. In our laboratory, Liu et al. [18] detected specific protein markers for the diagnosis of TB, and established a model based on the three biomarkers (2024, 8007, 8598 Da) to differentiate between patients with TB and other lung diseases by using surface enhanced laser desorption/ionization - time of flight - mass spectrometry (SELDI-TOF-MS). However, due to poor reproducibility of results, further study is needed for screening pulmonary TB biomarkers.

It has been shown that the serum miRNAs are stable and can not be easily degraded by enzymes or affected by temperature and time changes. It has also been demonstrated that miRNAs are resistant to acid and alkali [19], [20]. Recently, miRNAs have been intensively studied as new biomarkers for diagnosis and prognosis in various diseases such as, cancers, heart disease, diabetes, psychosis, and infectious diseases [21]–[25]. Liu et al. [26] identified miRNAs from fresh peripheral blood mononuclear cells (PBMCs) of 3 patients with smear-positive pulmonary TB and 3 matched healthy controls by using miRCURY™ LAN microRNA array (V.11.0). Analysis of the expression profile showed that expression of 30 miRNAs was significantly altered during active TB. Real-time PCR was performed and showed that miR-144* was significantly up-regulated in active TB patients, compared to healthy controls, and might play a role in the regulation of anti-TB immunity through modification of cytokine production and cell proliferation of T cells. Wang et al. [27] used a human miRNA microarray (Agilent Human miRNA microarray platform, version 3) to perform the miRNAs assay in PBMCs from 6 patients with active TB, 6 donors with latent TB infection (LTBI), and 3 healthy individuals. Seventeen miRNAs were differentially expressed between the three groups (*P*<0.01). The authors used a SYBR-Green miRNA real-time qPCR analysis of miRNA from PBMCs from 23 patients with active TB, 23 subjects with LTBI, and 15 healthy controls. They observed that hsa-miR-424 and hsa-miR-365 exhibited increased expression levels in samples from active TB versus healthy control groups. Using the predicted target genes, the regulatory networks of miRNAs was constructed. The regulatory network revealed that 17 miRNAs and their target genes may be involved in the transition from latent to active TB. Wu et al. [28] measured the expression profile of miRNA under purified protein derivative (PPD) challenge using microarray analysis (Agilent’s human miRNA microarray, version 3) in PBMCs isolated from 4 active TB patients and 3 healthy controls. Fourteen of 866 human miRNAs exhibited at least 1.8-fold difference in the ratio of expression level before and after stimulation with PPD between the active TB and healthy controls groups. The qRT-PCR was employed in PBMCs isolated from active TB patients (n = 21) and healthy controls (n = 19). The results showed that miR-155 and miR-155* were differentially expressed between the two groups. All above studies focused on the PBMCs miRNAs from pulmonary TB patients, and different miRNAs have been found using different microarray platforms. However, it is suggested that specific miRNAs do exist in pulmonary TB patients. Because miRNAs in serum are more stable, the miRNA profile in serum is more suitable to reflect disease states in the clinical laboratory [19].

Fu et al. [29] employed specific miRCURY LNA microarray platform (miRCURY LNA array, V.16.0) to compare the levels of circulating miRNAs between patients with active pulmonary TB and matched healthy controls. 91 miRNAs with significantly altered expression were identified, of which 59 miRNAs were over-expressed and 33 miRNAs were under-expressed in the TB serum. The real-time PCR analysis validated two differentially expressed miRNAs in patients with active pulmonary TB, compared to the healthy controls. The analysis of the ROC curve showed that up-regulated miR-29a could discriminate TB patients from healthy controls with reasonable sensitivity (83%) and specificity (80%). The expression of miR-93* was present in higher abundance in the TB serum than that of the healthy controls, however, there was no significant difference for the sputum levels of miR-93* between TB patients and controls. Qi et al. [30] showed that 97 miRNAs were differentially expressed in 20 pulmonary TB patients sera compared with 20 healthy controls by using the TaqMan Low-Density Array (TLDA v2.0, Applied Biosystems, CA, USA). Following qRT-PCR confirmation, expression levels of miRNAs in serum samples from 30 patients with active TB, 65 healthy volunteers and 60 patients with Bordetella pertussis, varicella-zoster virus and enterovirus were analyzed. The three miRNAs (miR-361-5p, miR-889 and miR-576-3p) were shown to distinguish TB infected patients from healthy controls and other microbial infections. It was suggested that the three miRNAs have great potential to serve as non-invasive biomarkers for early detection of pulmonary TB infection. But, due to the lack of specific clinical manifestations in patients, pulmonary TB may resemble other pulmonary diseases [31]. Common lung diseases, such as pneumonia and lung cancer should be set up as differential diagnosis groups. There were no differential diagnosis groups in the above mentioned study. So, screening of miRNAs can not rule out other lung diseases. In addition, due to different methods and samples, there is no overlap in miRNAs found in other studies. Meanwhile, in the above studies, the samples were small and the microarray detection was not stable, leading to poor reproducibility of results.

Profiling of miRNA in serum has been used as a diagnostic marker for pulmonary TB. According to the requirements for biomarkers in diseases, we speculated that 1–2 miRNAs used as diagnostic markers had low specificity [9], [32]. And due to individual differences in different patients, 1–2 miRNAs were extremely unreliable. Therefore, all above studies were insufficient to describe diagnostic markers for the early diagnosis of pulmonary TB. Our study is the first to detect serum miRNAs in pulmonary TB patients by using Solexa sequencing, which can detect any small RNA between 17–35 nucleotides. The technology can be used to discover new miRNAs and detect small changes in the length and sequence of known miRNAs. The advantages of Solexa sequencing are high speed, low cost and deeper coverage [5], [33]. In this study, specific miRNAs for pulmonary TB were screened from 326 serum samples (including differential diagnosis groups), and a combination of miRNAs were used as markers for the diagnosis of pulmonary TB with reasonable reliability.

In this study, six serum miRNAs (hsa-miR-378, hsa-miR-483-5p, hsa-miR-22, hsa-miR-29c, hsa-miR-101 and hsa-miR-320b) were shown to be specific for pulmonary TB patients, compared to non-TB patients (healthy controls, and pneumonia, lung cancer and COPD patients). Following ROC curve analysis for a single serum miRNA and a combination of the six miRNAs, the results showed that a combination of six serum miRNAs could discriminate pulmonary TB patients from healthy controls with reasonable sensitivity (95.0%) and specificity (91.8%), compared to a single serum miRNA. Analysis of genes predicted to be targeted by the obtained miRNAs revealed that 176 genes were related to pulmonary TB, and most encoded proteins were involved in the MAPK signaling, Wnt signaling, and TGF-beta signaling against *Mtb* infection that were useful to clarify the pathogenesis of pulmonary TB and medication used to treat pulmonary TB. Our results suggest that the miRNAs may regulate *Mtb* infection by signaling pathways and a combination of six miRNAs can serve as potential biomarkers for the diagnosis of pulmonary TB. Our results will provide useful facts and data to the understanding of diagnosis of pulmonary TB.

## Supporting Information

Figure S1 **A flow chart of the experimental design.**
(TIF)

Table S1 MiRNAs primers for qRT-PCR.
(DOC)

Table S2 Novel miRNA precursor candidates of miRNAs in pooled serum samples from pulmonary TB patients and healthy controls by Solexa sequencing analysis.
(DOC)

Table S3 Differentially expressed miRNAs in pulmonary TB serum samples compared to healthy controls determined by Solexa sequencing analysis.
(DOC)
