# Supplementary figures and images for: Screening and Identification of Six Serum microRNAs as Novel Potential Combination Biomarkers for Pulmonary Tuberculosis Diagnosis
Source: PLoS One. 2013 Dec 5;8(12):e81076. doi: 10.1371/journal.pone.0081076 (PMC3857778; doi:10.1371/journal.pone.0081076)

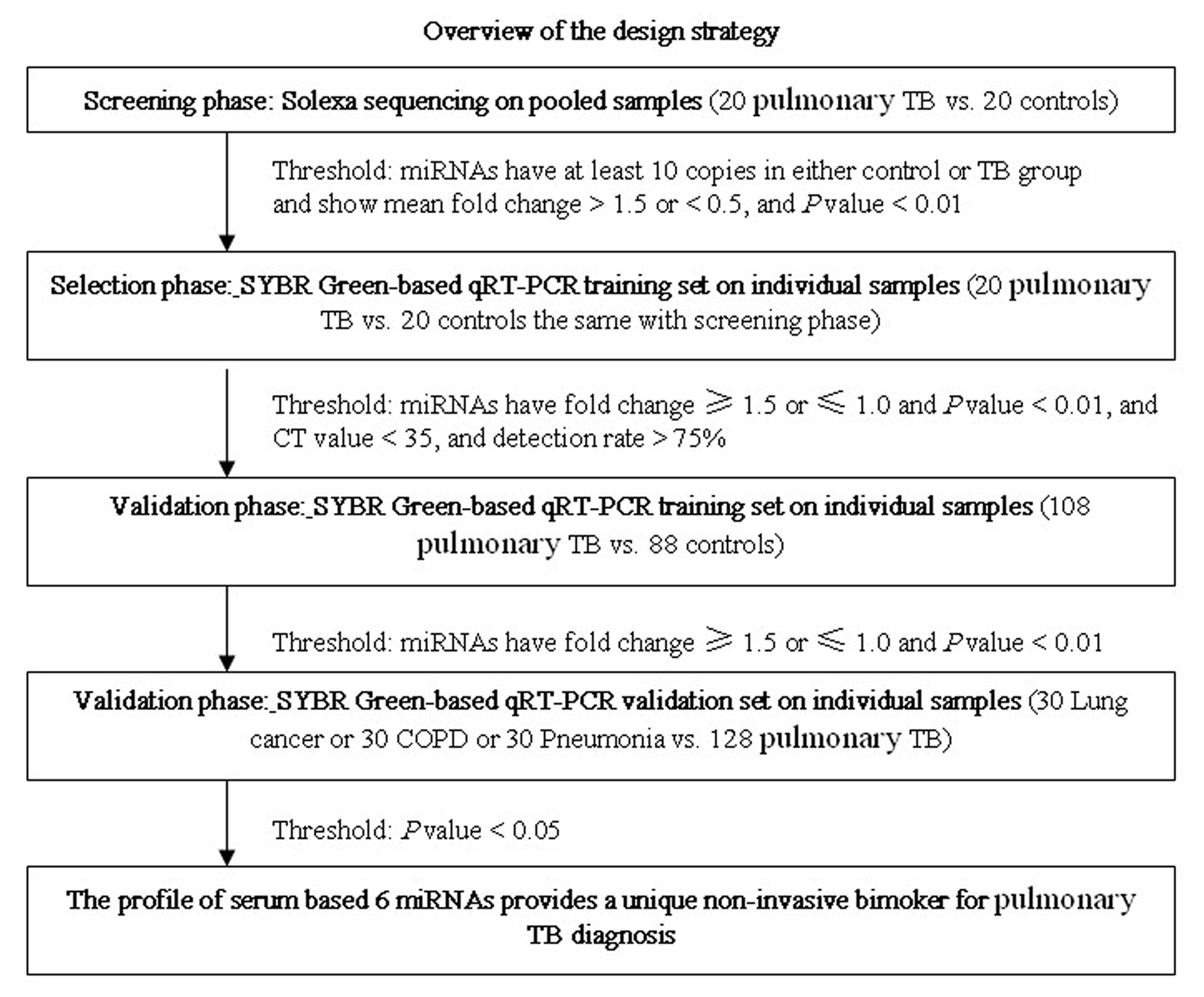

Supplement: Figure S1 — A flow chart of the experimental design. (TIF) [file pone.0081076.s001.tif]
