# Supplementary material for: Screening and Identification of Six Serum microRNAs as Novel Potential Combination Biomarkers for Pulmonary Tuberculosis Diagnosis
Source: PLoS One. 2013 Dec 5;8(12):e81076. doi: 10.1371/journal.pone.0081076 (PMC3857778; doi:10.1371/journal.pone.0081076)
Supplement: Table S1 — MiRNAs primers for qRT-PCR. (DOC) [file pone.0081076.s002.doc]

**Table S1** MiRNAs primers for qRT-PCR

| **mRNAs, miRNAs (*)** | **forward primer sequence** |
| --- | --- |
| hsa-miR-122 | 5’-CGCGGTGGAGTGTGACAATG-3’ |
| hsa-miR-146b-5p | 5’-ATGGCCTGAGAACTGAATTCC-3’ |
| hsa-miR-148a | 5’-CGTCAGTGCACTACAGAACTTTG-3’ |
| hsa-miR-181a-2* | 5’-ATCTGTCACCACTGACCGTTG-3’ |
| hsa-miR-22 | 5’-CGGAAGCTGCCAGTTGAAGA-3’ |
| hsa-miR-29c | 5’-GGCGGTAGCACCATTTGAA-3’ |
| hsa-miR-320c | 5’-ATGCCAAAAGCTGGGTTGA-3’ |
| hsa-miR-378 | 5’-GATAATACTGGACTTGGAGTC-3’ |
| hsa-miR-483-5p | 5’-TCTCGGAAGACGGGAGGA-3’ |
| hsa-miR-93 | 5’-CGCGGCAAAGTGCTGTTC-3’ |
| hsa-miR-1 | 5’-GGGCGCTGGAATGTAAAGAAG-3’ |
| hsa-miR-101 | 5’ -GGGTACTGTGATAACTGAAGG-3’ |
| hsa-miR-15a | 5’ -CGCCTAGCAGCACATAATGG-3’ |
| hsa-miR-26a | 5’ -CGCGCATTCAAGTAATCCAGG-3’ |
| hsa-miR-320b | 5’-AAAAGCTGGGTTGAGAGGGCA-3’ |
| hsa-miR-16 | 5’-CGCGCTAGCAGCACGTAAAT-3’ |

*, the opposite arm of the precursor.
