# Supplementary material for: Screening and Identification of Six Serum microRNAs as Novel Potential Combination Biomarkers for Pulmonary Tuberculosis Diagnosis
Source: PLoS One. 2013 Dec 5;8(12):e81076. doi: 10.1371/journal.pone.0081076 (PMC3857778; doi:10.1371/journal.pone.0081076)
Supplement: Table S2 — Novel miRNA precursor candidates of miRNAs in pooled serum samples from pulmonary TB patients and healthy controls by Solexa sequencing analysis. (DOC) [file pone.0081076.s003.doc]

**Table S2** Novel miRNA precursor candidates of miRNAs in pooled serum samples from pulmonary TB patients and healthy controls by Solexa sequencing analysis.

| **Sample** | **Number of unique miRNA candidates** | **Number of total**  **miRNA candidates** |
| --- | --- | --- |
| Healthy controls | 19 | 17438 |
| Pulmonary TB | 13 | 4393 |
