# Supplementary material for: Screening and Identification of Six Serum microRNAs as Novel Potential Combination Biomarkers for Pulmonary Tuberculosis Diagnosis
Source: PLoS One. 2013 Dec 5;8(12):e81076. doi: 10.1371/journal.pone.0081076 (PMC3857778; doi:10.1371/journal.pone.0081076)
Supplement: Table S3 — Differentially expressed miRNAs in pulmonary TB serum samples compared to healthy controls determined by Solexa sequencing analysis. (DOC) [file pone.0081076.s004.doc]

**Table S3** Differentially expressed miRNAs in pulmonary TB serum samples compared to healthy controls determined by Solexa sequencing analysis.

| **miRNAs-names** | **Copy number**  **in pulmonary TB** | **Copy number**  **in healthy controls** | **Fold change** (**pulmonary TB /healthy controls)** |
| --- | --- | --- | --- |
| **Up-regulation in pulmonary TB** | | | |
| hsa-miR-122 | 226454 | 57478 | 3.939838 |
| hsa-miR-146b-5p | 4244 | 1683 | 2.521687 |
| hsa-miR-148a | 1554 | 16 | 97.125 |
| hsa-miR-181a-2* | 3866 | 24 | 161.0833 |
| hsa-miR-22 | 10806 | 2360 | 1.578813 |
| hsa-miR-29c | 2796 | 12 | 233 |
| hsa-miR-320c | 1917 | 766 | 2.502611 |
| hsa-miR-378 | 6669 | 25 | 266.76 |
| hsa-miR-483-5p | 9669 | 2073 | 4.664255 |
| hsa-miR-93 | 1370 | 297 | 4.612795 |
| **Down-regulation in pulmonary TB** | | | |
| hsa-miR-1 | 2044 | 9494 | 0.215294 |
| hsa-miR-101 | 193 | 2150 | 0.089767 |
| hsa-miR-15a | 10 | 2648 | 0.003776 |
| hsa-miR-26a | 13 | 3132 | 0.004151 |
| hsa-miR-320b | 883 | 7925 | 0.11142 |
